# Adolescents’ experiences of a school-based health promotion intervention in socioeconomically advantaged and disadvantaged areas in Sweden: a qualitative process evaluation study

**DOI:** 10.1186/s12889-023-16581-z

**Published:** 2023-08-25

**Authors:** Lisette Farias, Gisela Nyberg, Björg Helgadóttir, Susanne Andermo

**Affiliations:** 1 Department of Neurobiology, Care Sciences and Society, Division of Nursing, Karolinska Institutet, Huddinge, 141 83 Sweden; 2 Department of Sport Science, The Swedish School of Sport and Health Sciences, Lidingövägen 1, Stockholm, 114 33 Sweden; 3 Department of Global Public Health, Karolinska Institutet, Stockholm, 171 77 Sweden; 4 Department of Clinical Neuroscience, Karolinska Institutet, Stockholm, 171 77 Sweden

**Keywords:** Health inequalities, Health promotion, School intervention, Young people

## Abstract

**Background:**

Adolescence is a transition period in which positive experiences of physical activity have the potential to last into later adulthood. These experiences are influenced by socioeconomic determinants, leading to health inequalities. This study aims to explore adolescents’ experiences and participation in a multi-component school-based intervention in schools located in socioeconomically advantaged and disadvantaged areas in Sweden.

**Methods:**

A qualitative design was used to evaluate how participants experienced the intervention. The intervention was a multi-component school-based intervention. It was conducted in six schools (four control and two intervention schools) with a total of 193 students and lasted one school year. It was teacher-led and consisted of three 60-minute group sessions per week: varied physical activities, homework support with activity breaks, and walks while listening to audiobooks. In total, 23 participant observations were conducted over eight months and 27 students participated in focus groups. A content analysis was conducted.

**Results:**

The results describe a main category ‘Engaging in activities depending on socioeconomic status’ and three generic categories: 1. Variations in participation in PA together with classmates and teachers; 2. Variations in engagement in PA after school; and 3. Differences in time and place allocated to do homework and listen to audiobooks. These categories illustrate how participants looked forward to the physical activities but used the time spent during the walks and homework support differently depending on how busy they were after school. Frequently, those who were busiest after school were also those from the advantaged area, and those who had little to do after school were from the disadvantaged area.

**Conclusion:**

Socioeconomic factors influence participants’ possibilities to engage in the intervention activities as well as how they use their time in the activities. This study showed that it is crucial to support adolescents’ participation in physical activities by providing structure and engaging well-known teachers in the activities, especially in schools located in disadvantaged areas.

## Background

Adolescents face many challenges, including increasing academic expectations and the physical changes associated with maturation [1]. Worldwide, the strongest determinants of adolescent health are structural factors, such as national wealth, income inequalities, and access to education [2]. International studies have shown constant or growing inequalities in adolescent self-rated health associated with adolescents’ parental affluence [3–5]. Parental socioeconomic status is persistently linked to educational attainment [6] with adolescents from high-income families often residing in advantageous school districts and performing better than those from less affluent districts [6, 7]. Moreover, socioeconomic differences in parental and contextual factors influence adolescents’ physical activity (PA) [8]. It is known that adolescents from families with lower socioeconomic status are more likely to be less physically active [9, 10] and reside in areas with fewer opportunities to be active outdoors [11]. For example, Schmengler et al. [12] reported that higher family affluence was strongly associated with higher levels of adolescent PA across thirty-two different countries, including Nordic countries. Despite living in welfare states, adolescents in Nordic countries experience disparate socioeconomic conditions that have changed over time due to economic crises and globalization [1]. Over the last few decades, OECD data show that income inequality has grown faster in Sweden compared to other countries [13].

When focusing on adolescent health within the Swedish context, some issues should be addressed to understand where evidence about differences in adolescents’ educational achievement and PA stands today. National results indicate that the impact of socioeconomic background on learning has increased over the last years [14, 15]. For instance, the latest results in grades four and eight showed that Swedish adolescents from a prosperous socioeconomic background, measured by the availability of home resources for learning (e.g., number of books, access to internet, their own room, and a desk), achieved better academic results than those from a less prosperous socioeconomic background. Corresponding differences were seen in all the Nordic countries for results in both mathematics and natural science, with advantaged adolescents outperforming their less advantaged peers. In Sweden, the difference also tended to increase over time, above all in grade eight [16].

Educational achievement has also been significantly associated with adolescents’ participation in organized extra-curricular physical activities in the Swedish context (i.e., the more affluent the adolescents’ background, the more likely they are to get good grades and participate in organized activities) [17]. This is in line with the results of the first national survey of PA and sedentary time among Swedish adolescents, which indicated that adolescents, and especially girls, with low socioeconomic status have the lowest levels of PA [18]. This might be due to economic factors related to the costs of sports equipment and membership fees required to participate in organized PA [19]. In a study in a Swedish community with low socioeconomic status, lack of financial support from their parents was identified as a factor that hindered adolescents’ participation in organized PA [20].

When attempting to address health inequalities among adolescents, the school has long been defined as the ideal setting for health promotion interventions without having to discriminate based on socioeconomic background [21–23]. A study in Norway across thirty secondary schools suggested that school-based PA interventions were viable models to increase academic performance among adolescents [24]. Nonetheless, most school-based interventions tend to treat PA and cognitive function as unrelated processes, even though a more integrated approach is recommended for more effective health and learning outcomes, an approach that would also benefit adolescents from districts with low socioeconomic status [25]. Moreover, it is important to understand how socioeconomic factors influence the implementation of interventions. Only a few qualitative studies have reported school-based PA interventions targeting adolescents from socioeconomically disadvantaged areas [26, 27]. More research on the challenges and complexity of delivering successful interventions is needed to reduce health disparities, especially across multiple socioeconomic contexts [23].

Process evaluations are required to ensure that interventions are reaching the intended population. However, most process evaluations exploring adolescents’ experiences of school-based PA interventions are not set up to explore socioeconomic differences [28–31]. Some studies report process evaluations of school-based PA interventions targeting only adolescents with low socioeconomic status [32–34] but they do not account for socioeconomic differences related to school districts. Although it is well-known that process evaluations are critical in exploring how contextual factors can influence how an intervention is delivered and received [35], there is a lack of studies reporting how schools’ socioeconomic backgrounds may influence adolescents’ participation in school-based interventions. This study aims to explore adolescents’ experiences and participation in a multi-component school-based intervention consisting of varied physical activities, homework support, and walks while listening to an audiobook, in schools located in socioeconomically advantaged and disadvantaged areas in Sweden.

## Methods

### Design

An ethnographic approach [36] was used since it has been identified as being particularly well suited to the purpose of capturing systems’ complexity in intervention evaluation [37]. This approach consisted of participant observations with field notes [38, 39], and focus groups [40, 41]. Participant observations were chosen to follow the adolescents participating in the intervention closely and gain complementary insights and understandings through engagement over a long time span [42]. During ethnographic fieldwork and observations, the first and the last author noticed differences among adolescents attending the different socioeconomic school districts in terms of participation and use of the time dedicated to the intervention’s different components. Hence, focus groups were conducted with separate groups of adolescents from the different schools to provide a more comprehensive evaluation of the potential influence of the schools’ socioeconomic contexts on the participants’ experiences of the intervention. A content analysis [43] was conducted. The study is reported according to COREQ [44].

### Setting

This study is part of a process evaluation of an intervention (a cluster randomised controlled trial). The overall aim of the intervention was to develop an effective universal multi-component school-based intervention targeting varied physical activities, homework support with activity breaks, and walks while listening to audiobooks during an extended school day and evaluate its effects on mental health, physical health, cognitive function as well as academic performance. The intervention components were (1) different PA (e.g., dance, ball games), (2) homework support with activity breaks, and (3) walking and listening to an audiobook. All components were designed to be carried out in groups, consisting of 60-minute activities, three times a week, and led by teachers. The integration of the three components in the school schedule was designed to enhance inclusion and equity for all students regardless of their socioeconomic background by being cost-free for students and including all components in the school schedule.

The intervention had been developed through a preparation phase consisting of workshops with students, school principals, and teachers to gather information about potential barriers and facilitators related to implementation (not yet published). The workshops provided information about implementation strategies (e.g., times that would be most suitable to integrate the components in the school’s schedule), ways of motivating students to participate in the intervention, and types of PA that could be used and were different from Physical Education activities. Furthermore, the intervention was grounded in two theoretical approaches. The first is a Person-In-Environment perspective following Bronfenbrenner’s Socioecological model [45], which highlights how various layers of society and institutions such as schools along with the interactions between individuals and their interpersonal life are interrelated. The second is the Self-determination Theory [46] which was used to understand adolescents’ motivation toward the intervention’s components.

The intervention schools scheduled the activities and decided the most suitable times to integrate the intervention components (e.g., before the first class, before or after lunch, and after the last class). The teachers involved in the intervention were well-known by the students and led all the components. In some cases, the PA components were led by teacher assistants and in others by Physical education teachers. All teachers involved in the intervention met the research team and received instructions and support on how to conduct the three components.

Half of the schools included in the intervention were randomised to participate in the intervention and half were in the control group. Originally, eight schools were recruited but two withdrew before the intervention started. A total of two intervention schools and four control schools were recruited. The participation rate of adolescents in the data collection and evaluation process was 90% (*n* = 193). Of these participants, 51% (*n* = 89) were in the intervention group and 49% (*n* = 85) were in the control group. The intervention was implemented during one school year, from September 2021 to June 2022. All participating students were invited to pre and post-intervention measurements. The measurements consisted of questionnaires about mental health, motivation to PA, screen time, and health behaviours. Cognitive function (working and episodic memory) was measured with computer-based tests, PA was measured by accelerometry, cardio-respiratory fitness with a step test, academic performance by grades, and body weight and height were measured.

The process evaluation was conducted in both intervention schools with five classes (three in one school and two in the other school). These schools happened to be located in two different districts in Stockholm, one with low and the other with high socioeconomic status. The socioeconomic differences in school districts emerged as an evident result in the qualitative explorative process evaluation, and the aim of the study was finetuned to further explore these differences. Socioeconomic status comprises material and social resources such as income, educational level, and class as socially mediated and central components of the social context [47]. The school in the area with low socioeconomic status belongs to a district in north-western Stockholm with a large proportion of persons with an immigrant background, with a higher proportion reporting a limiting longstanding illness, lower education level, and a lower proportion of working-age adults who are gainfully employed [48]. The adult population residing in this area shows lower PA levels and a greater proportion of daily smokers than the population living in the areas with high socioeconomic status [48]. The school representing high socioeconomic status is located in an area with low obesity prevalence in the adult population and a high percentage of adolescents belonging to households with Swedish backgrounds and high buying power [49].

### The school context

The Swedish school context has a long tradition of promoting equality and opportunity. Yet, differences in academic attainment between schools have increased steadily since the early 1990s [14, 15]. This is mainly due to students from advantaged backgrounds perform better than students from less advantaged backgrounds who often reside in more segregated areas [7]. These differences have also been associated with differences in resources, teachers’ skills, and the school’s ability to manage its teaching resources and equalize the opportunities between different groups of students [50]. Schools whose students come from less advantaged backgrounds have higher teacher-pupil ratios compared to schools whose pupils come from more advantaged backgrounds. However, teacher turnover is higher at schools with students from less advantaged backgrounds [50].

### Selection of participants

Adolescents in grade eight (14–15-year-old boys and girls) from the two intervention schools were chosen for the observations. All five classes were included. For the focus groups, a face-to-face verbal invitation was made by the first author to each class, and only those students interested in participating contacted the teachers involved in the intervention to receive written information about the study. The same teachers collected consent forms signed by the participants’ guardians. Each participant received a cinema ticket as compensation for their time in the focus groups.

### Data collection

#### Participant observations and field notes

A research assistant and the last author assisted the first author in conducting participant observations. The observations were conducted 1–2 times each week with all classes, at different times and days during the school week, from October 2021 to May 2022. Participant observation in an ethnographic study entails varying degrees of participation. In this study, moderate participation [39] was used, which means that the researcher did not take initiatives directed at the adolescents but was sensitive to changes over time. Observations provide an opportunity to follow adolescents and obtain a complete picture of the schools’ physical, cultural, and socioeconomic environment. For example, it allowed researchers to gain insights into how the weather affected the activities, who interacted the most and the least, and where the adolescents were walking.

To be able to understand how the activities were experienced by the adolescents, it was important that the researchers (adults, females) were present at the different activities and became accepted by the participants. For this purpose, the first author introduced herself to staff and adolescents on several occasions, answered all questions regarding her role, and dressed casually to avoid distracting adolescents from the activities being observed [51]. To reflect on her position and the information gathered, field notes were taken during and after the observations and consisted of detailed notes describing the interactions among adolescents and with the teachers leading the activities. A grid elaborated based on an ethnographic approach [36, 39] was used to document the sessions. Field notes were used as complementary to participant observations and supported researchers’ reflexivity [52].

#### Focus groups

Five focus groups with four to six students in each were conducted after the intervention had ended (May 2022). All students involved in the two intervention schools were invited to participate in the focus groups and those that were interested and provided informed consent were recruited. A total of 27 students from the five classes involved in the intervention were recruited (18 boys and 9 girls). The students represented a varied group in terms of attitude towards the intervention (i.e., positive, and negative attitudes) and level of PA (i.e., some were enrolled in extra curriculum activities while others were not enrolled but were very engaged in the PA provided by the intervention, and others were more active when the PA suited their interest). Two focus groups were conducted at the school in the advantaged area (*n* = 14) and three focus groups at the school in the disadvantaged area (*n* = 13). Each focus group lasted for about 60 to 75 min and was conducted in Swedish. The questions posed in the focus groups aimed at gaining a deeper understanding of the students’ experiences of the intervention based on their perceptions and how contextual and organizational factors might influence their participation in the different components of the intervention. The first and last authors were present in all focus groups, where one led the groups while the other took notes and observed. Since the focus groups were intended to capture participants’ experiences, it was important to ensure that all students had the opportunity to express their opinion, and therefore participation guidelines, such as waiting for one’s turn to talk or raising one’s hand to talk, were established. Yet, students interacted freely in the groups, for example commenting on each other’s experiences by shaking their heads or making affirmative sounds, they also commented on each other sometimes and these discussions were not interrupted by the facilitator unless the discussion escalated by introducing a new question or giving space for another student to respond. These confirmations and contradictions were transcribed and integrated into the analysis.

To facilitate students’ participation, the researcher leading the focus groups was the same person who conducted the observations and followed the students during the intervention. A semi-structured guide with open questions was used. Examples of questions included: Can you tell us about the intervention? What worked well/not so well? Which parts did you like the most/least? Probing questions were used to clarify specific content or ask for examples of situations that participants mentioned. This approach was used to minimize leading questions. Focus groups were audio recorded and notes were taken by the last author during and after the focus groups.

### Data analysis

To ensure consistency with an ethnographic approach [36], the first author led data collection and analysis as an iterative process to increase understanding of the observations made in the context. After the data collection was completed, the first author reviewed the field notes from the observations and focus groups several times to ‘obtain a sense of the whole’ ([54], p. 108). At this point, it was noticed by the researchers that the data provided valuable insights about differences in students’ participation in the intervention related to their socioeconomic school districts. Then, the analysis was primarily focused on identifying similarities and differences in the empirical data, particularly on the potential differences in different groups’ experiences of each component of the intervention due to contextual factors. Using content analysis [43], the coding and re-coding process was led by the first author who then grouped similar codes into tentative categories. These categories were thoroughly examined by the first and the last author together. This process was repeated a few times until no additional categories or possibilities to combine them were identified. These revised categories were finalized after consensus was reached among all authors to validate the process. All authors are female researchers with previous experience and training in qualitative research, process evaluation, or PA school-based intervention with children and adolescents. Since all authors have Swedish as a first or second language, quotes used in the findings were translated from Swedish to English after the analysis was completed. In the presentation of the findings, the participants are anonymized by using pseudonyms.

### Ethical considerations

Participants and their parents were informed about the study, that participation was voluntary, and that they could leave the study at any time. All methods were carried out in accordance with Swedish regulations. Ethical approval for this study was obtained from the Swedish Ethical Review Authority (Dnr 2021 − 00911). Informed consent to participate was obtained from all participants’ legal guardians.

## Results

**Fig. 1:**
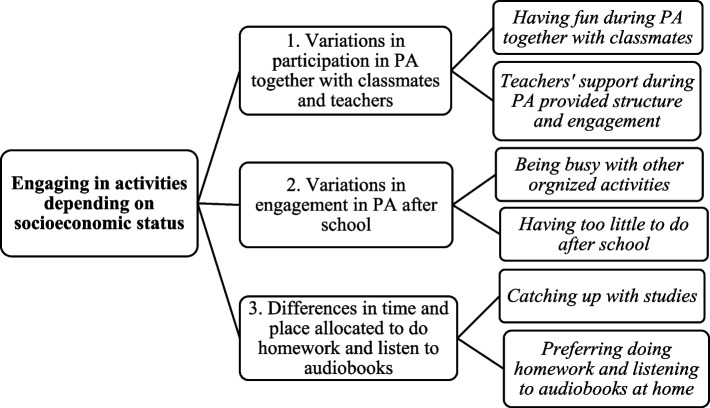
Main Category, generic categories, and sub-categories’ structure

A main category ‘Engaging in activities depending on socioeconomic status’ and three generic categories were found in the analysis (See Fig. 1). The three categories were: 1. Variations in participation in PA together with classmates and teachers; 2. Variations in engagement in PA after school; and 3. Differences in time and place allocated to do homework and listen to audiobooks. The three categories are related to each other and within the main category by illustrating how socioeconomic factors influence adolescents’ opportunities to engage in the intervention. Six sub-categories related to specific components of the intervention and variations in school groups’ experiences and use of the time allocated to the different activities. All names used are pseudonyms.

### Engaging in activities depending on socioeconomic status

#### Variations in participation in PA together with classmates and teachers

##### Having fun during physical activities together with classmates

Students from both schools described that the physical activities were fun since they provided an opportunity to do something together with all their classmates. During the observations, those participating in the physical activities often commented to the teachers and researchers involved in the observations that they looked forward to the activities and have fun with other classmates with whom they did not usually spend time together. Having fun during the physical activities made them go home with a good feeling.

It was quite fun because we needed to talk to each other, we had fun together. Not all by ourselves. It was fun to end the day with some fun activities. It was always fun to go home because you went home with good energy (Ida, school in disadvantaged area (DA)).

Although the group from the school located in the advantaged area participated in the physical activities, it was observed that they came late, went home early, or dropped out of the activities over time. It was also observed that the group from the school located in the disadvantaged area often participated in the activities from start to finish and instead had difficulties ending the activities. The teachers at both schools usually offered different activities from which students could choose, such as volleyball, strength training, dance, and table tennis, yet the group from the school in the disadvantaged area showed to be more motivated throughout the whole intervention period.

I was quite shocked because there were a lot of people who joined [PA]. Everyone was there, both girls and boys, it was the same. There was no ‘but I don’t want to play this’ and then you can always choose between different things. So, it was always fun, so you wanted to come to class. (Ida, DA)

### Teachers’ support during physical activities provided structure and engagement

The activities were experienced as fun not only because all classmates were engaged but also because teachers participated in the activities, sometimes playing with the students. In the disadvantaged area, the students emphasized the value of having teachers who were actively engaged in the activities. Teachers’ involvement contributed to maintaining the students’ positive motivation and participation.

They [the teachers] are active, they participate, they are with us, they play with us, they have fun with us, they help us divide into teams. They solve everything. (Erik, DA)

It was also observed that teachers from the school in the disadvantaged area helped students to structure the activities to avoid conflicts among students and support the group in deciding which activities were most appealing to them. The teachers’ participation in the physical activities was also described as contributing to a good school environment/atmosphere.

Students from the school located in the advantaged area also related the participation of teachers to having fun in the activities, but especially important was having teachers who knew the students so that they could modify the activities based on the group’s needs.

[The adaptation] I think it came from the teacher (name) understanding that after a long day, you might not have the energy to run around the gym hall for an hour and that a walk might be enough. (Emanuel, school in advantaged area (AA))

#### Variations in engagement in PA after school

##### Being busy with other organized activities

Although both groups of students expressed enjoying the physical activities and walks while listening to audiobooks, some students also expressed that they were already busy with other organized physical activities after school. This was mainly observed among the students in the school in the advantaged area, who often mentioned that they participated in other organized activities after school, such as football, volleyball, and horseback riding.

There was just more to do really because after my training I’ll just [have time to] study and then rest. (Simon, AA)

For example, the group of students, mainly from the school in the advantaged area, often commented during the walks that they felt a bit stressed when they had to stay at school longer to participate in the activities provided by the intervention. When having a longer school day, these students experienced that they could feel stressed as they needed to rush home, eat something quickly and rush again to get to their other activities on time.

Instead of it being something that helped you, it created extra stress because you didn’t have time to do what you were supposed to do. (Wilhelm, AA)

The group of students from the school in the advantaged area also emphasized that because they have access to organized activities after school, the intervention would be most beneficial for those students that do not have the same resources or are not as physically active.

It’s a good idea for those who may not exercise as much. They get this extra movement, so they get the exercise that the body needs in addition to sports. (Wilhelm, AA)

### Having too little to do after school

On the other hand, most students from the school located in the disadvantaged area described having little to do at home. During the observations, it was evident that this group of students did not want to end the activities and go home; they usually stayed until the teachers had to explicitly ask them to leave the school gym where the activities were conducted. Some of them commented that at home, they sometimes got bored. They usually just played with their mobile phones and waited for the next school day.

I think it’s great [extra activity at school] because you don’t have to go home and sit and play on your mobile phone and computer. Exercising is more fun than playing video games [instead] you play with your friends and the teacher. (Hugo, DA)

[At home you have] nothing special, you just go home, do what you want to do and then wait until the next day. (Linus, DA)

#### Differences in time and place allocated to do homework and listen to audiobooks

##### Catching up with studies

Students in both schools were clearly motivated by the homework support and walks listening to audiobooks. Yet, it was observed that they used the time allocated to these activities differently. For example, more students in the advantaged area, and especially those who had other activities after school, used the time provided by homework support to study and do their homework. They maintained that it was good to be able to use the time to catch up with their studies:

It [homework support] was good, I think, especially when we had certain exams that day, so you could get extra time to study during school hours. (Emanuel, AA)

Students from the school in the advantaged area also used their time during the walks to listen to audiobooks, especially if they needed to catch up with their reading in certain school subjects.

I thought that the audiobook was very positive because there are books that you read at school, so you can also get schoolwork done during the walks in some way by listening to it. It was very practical, you could take a walk and then listen to those books while you catch up. (Robert, AA)

##### Preferring doing homework and listening to audiobooks at home

Students in the school in the disadvantaged area sometimes used the time to study during homework support, but it was more frequently observed that they preferred to use their time during homework support to socialize. They expressed that they preferred to study and do their homework at home. The main reason they mentioned was that they had nothing to do after school, such as other organized activities.

My homework, I don’t do it at school, I do it at home. Because at home, I have nothing to do at home. You usually get tired of your mobile phone when you’re sitting at home. And then if you have nothing to do, you can study. (Hugo, DA)

It was also noticed during the observations that students in the disadvantaged school more often used their time during the walks to socialize and talk. This was clarified by the students during the focus groups who described that they could not concentrate during the walks since most of their classmates were talking to each other. Therefore, most of them used the audiobook account downloaded to their mobile phones to listen at home, on the bus, or when walking alone.

I usually listen on the bus. Afterwards, when you’re alone, then you can become better [at listening]. (Lennart, DA)

## Discussion

This study explored how adolescents attending schools in areas with different socioeconomic statuses experienced and participated in a multi-component school-based intervention consisting of (a) different physical activities (PA), (b) homework support with activity breaks, and (c) walks while listening to audiobooks. The findings showed that although both groups of students described having fun during the intervention, their participation in PA varied. It was observed that those from the school in the disadvantaged area often had difficulties ending the activities, while students from the school in the advantaged area tended to come late or leave early, probably due to other extra-curricular engagements. In particular, the findings illustrated how participants used the time provided by the intervention components in different ways based on their possibilities to participate in other organized activities after school. The ways participants experienced the intervention are described in three categories: (1) Variations in participation in PA together with classmates and teachers; (2) Variations in engagement in PA after school: and (3) Differences in time and place allocated to do homework and listen to audiobooks.

A few previous studies have reported process evaluations of school-based interventions for adolescents in disadvantaged communities or from backgrounds with low socioeconomic status. In these evaluations, problems with intervention delivery such as lack of dissemination about the activities, limited space for the activities, and dealing with behavioural problems and inclement weather are described [32–34]. Yet socioeconomic differences among schools and how socioeconomic factors may affect responses to the intervention are less explored. This study furthers the understanding of how socioeconomic factors may influence adolescents’ engagement in school-based interventions by describing variations in engagement and the use of time allocated to intervention activities.

### Findings concerning possibilities to participate in PA with classmates and teachers

The category ‘Variations in participation in PA together with classmates and teachers’ and sub-categories ‘Having fun during PA together with classmates’ and ‘Teachers’ support during PA provided structure and engagement’ can be related to the importance of providing activities that support sociability and fun with peers outside of their usual friendship group. Having teacher-led PA with the whole class, allowed participants to choose among different activities and groups without having to take the initiative. This setting served as a ‘free zone’ in which adolescents could have fun together without peer social pressure or socioeconomic expectations. Socializing with others outside their friendship groups or between subgroups within the same class has been described by adolescents as key to the enjoyment of PA school-based interventions [29]. Peer group pressure is an important aspect of adolescence that can positively or negatively influence adolescents’ health behaviours [55]. Therefore, school-based interventions could benefit from involving teachers who are trusted by adolescents to lead and structure the PA sessions in a way that can reduce conflicts among groups [56]. Based on the observations from this study, teachers played a key role in facilitating the mixing of groups and connecting with other peers outside the friendship groups. When planning future interventions, it is recommended to involve teachers as mentors or health promoters to adopt role modelling and set structures that encourage adolescents to share and connect with peers in less socially and academically demanding settings.

### Having the possibility to participate in organized activities after school

The category ‘Variations in engagement in PA after school’ illustrates that some students had different possibilities to participate in activities after school. Although differences in access to organized activities can be related to adolescents’ interests and preferences, the sub-categories ‘Being busy with other organized activities’ and ‘Having too little to do after school’ show how these differences can be related to schools’ socioeconomic contexts. For instance, most participants in this study from the school in the advantaged area were involved in one or more organized activities after school. This group of students even mentioned that the PA component of the intervention would be most beneficial for those students who may not exercise as much. According to previous research in the Swedish context, participation in organized after-school activities is stratified by socioeconomic background, that is, participation rate increases in parallel with social class positions [17, 57]. This correlation between high- and low-income areas and high/low participation in organized activities is a well-recognized phenomenon in Nordic countries [58] and other wealthy countries [59]. Non-economic constraints could also contribute to this income gradient in organized activity participation as the result of parental time constraints (i.e., parents residing in lower income areas in Sweden have limited control over working hours) [60] and fewer organized activities and club sports available in less prosperous areas [57].

Living in disadvantaged areas can negatively impact adolescents’ educational achievements as well as their opportunities to participate in organized activities after school, leading to multiple social disadvantages [61]. Although adolescents’ participation in organized after-school activities in such areas is understudied [62], those studies focusing on this group consistently show that adolescents in areas with low socioeconomic status benefit more from participating in organized activities than those from areas with higher status [63, 64]. This is in line with the present results which showed that the students attending the school in the low socioeconomic area were more engaged in the physical activities. Further, the findings related to ‘Having too little to do after school’ are worrisome since they illustrate that lacking opportunities for organized activities can increase adolescents’ screen-time (i.e., mobile phone and computer use) and loneliness. For the planning of future interventions, it can be relevant to consider how adolescents spend their time outside school and if their participation in after-school activities presents barriers to participation in the intervention that can be modifiable.

### Using the time allocated to the activities differently

The current study also identified differences in how adolescents used the time allocated to intervention activities related to academic performance, such as homework support and walks while listening to audiobooks. The category ‘Variations in engagement in PA after school’ and sub-categories ‘Catching up with studies’ and ‘Preferring doing homework and listening to audiobooks at home’ illustrate how adolescents use the time allocated to the activities according to their own needs and possibilities. The ‘Catching up with studies’ sub-category emphasized how socioeconomic background can influence adolescents’ participation in activities that support academic performance. Although both groups of students participated in homework support and walking while listening to audiobooks, and considered academic performance relevant, they used this time differently. In line with previous studies based on ethnographic work in Swedish schools [61], students in the school located in the advantaged area seem to have high demands on their academic performance and the education provided. This was conveyed by students’ descriptions of homework support as highly valuable for their academic performance.

Conversely, students from the school in the disadvantaged area tended to use this time to socialize, preferring listening to audiobooks on their way to or from school and doing homework at home, probably to fill their time with meaningful activities. As such, students from the school in the advantaged area could take advantage of the homework support component of the intervention, while those in the disadvantaged area did not seem to benefit from the activities to the same extent. These findings illustrate that schools can both serve as equalizers of socioeconomic differences by providing support to all students, as well as contributing to such differences [65]. A lesson learned from the present study is that these two activities – homework support and walking while listening to audiobooks – entailed tasks that required concentration and were difficult to perform in groups for the students from the disadvantaged school. Providing more structure during homework support and allowing students to take walks individually could enhance a calmer atmosphere. It may also be beneficial to implement strategies that provide homework support with educational material that encourages students to engage in these activities actively rather than passively.

### Strengths and limitations of the study

The findings provide insights limited to two urban schools in Sweden and therefore transferability of the findings cannot be fully made; yet, involving schools located in socioeconomically advantaged and disadvantaged areas may be seen as positive. Implementing and adapting school-based programmes would not be possible without the exploration of schools located in different contexts. Although self-reported information from participants gathered through focus groups provided rich insights into their experiences, relying exclusively on self-report may lead to discrepancies between adolescents perceived and actual behaviour, and peer pressure may influence their responses. It is also possible that the cinema ticket offered as compensation for participants’ time may have limited the scope of the study by attracting those interested in the tickets. To overcome these limitations, the involvement of the same researchers during participant observations and focus groups was important [53]. The long-term engagement of the first author in the field assisted by the last author and a research assistant provided an opportunity to build a relationship with the adolescents so that they could share trustworthy accounts of their experiences. The use of observations, field notes, and focus groups for data collection is, therefore, a strength of this study. Investigator triangulation was enhanced by including the first and the last author in the data collection as well as the analysis of the transcripts [54]. The involvement of two other researchers in the data analysis who were not involved in the data collection to review and recheck the first categories was important to establish confirmability [53]. Reflexivity was ensured through peer debriefing among the first and last authors throughout the research process and by having constant discussions with the rest of the authors [52].

## Conclusion

The present study’s process evaluation provided relevant findings about students’ experiences of and participation in a multi-component health promotion intervention of adolescents attending schools located in different socioeconomic areas. The results describe how adolescents’ participation and use of their time in the activities were influenced by their socioeconomic context and access to organized after-school activities. The involvement of well-known teachers who provided structure and a safe setting was described by adolescents as an important factor in them having fun and trying out activities with classmates outside their friendship groups. The study also highlighted a need to provide more structure to homework support to enhance students’ active role and concentration, especially at schools in disadvantaged areas. Future research is needed to best develop strategies that meet the needs of students and school teachers located in schools in different socioeconomic contexts.

## Data Availability

The datasets analysed during the current study are available from the corresponding author upon reasonable request.
